# Monthly Summary

**Published:** 1899-03

**Authors:** 


### Monthly Summary.

INSTRUMENTS TO CARRY OUTSIDE.—One mouth-mirror (or two), to throw light.

One explorer (or two), to search cavities.

One large cutter (side); one large cutter (end); to break edges and to open cavity.

One (or two) long, smaller side cutter; one (or two) long hoe excavator; one short side cutter; one short hoe excavator; called excavators, to clean and partly excavate cavity.

One pair foil-tongs (always necessary).

One water syringe (convenient).

One knife file, or separating files (not always wanted).

One half-round, medium-cut file, for shortening bite.

One root instrument, for reaching into roots.

One gum-lance. (Have a sharp knife in your pocket).

Six small mouth-napkins, to dry with.

One wad of cotton or absorbent fibre. (Do not forget it!)

One spool floss silk or thread, to tie pledget in (sometimes).

One pair scissors, to cut silk.

One bottle sandarach varnish, to protect cotton filling.

One bottle nerve-destroying paste, to kill pulps.

One bottle 1, 2, 3 compound or cocain, or any good obtundent.

One small bottle toothache drops, to leave behind with patient.

On a chance of being obliged to extract an aching tooth, carry the following forceps :

One pair upper wisdom.
One pair under wisdom.
One pair upper bicuspid.
One pair under bicuspid.
One pair upper central and cuspid.
One pair under central and cuspid.
One elevator. (Use your own kind).

The wisdom forceps may, in an emergency, be used for all the molars; the bicuspid forceps for the canines. If not, use the central forceps for them and the remaining teeth.

Put all these implements into a small, neat hand-bag, with a clean towel, and, if room, add your thinnest office-coat, for comfort. Bring back all your instruments, etc., and your fee, if you can get it—cash.—J. T. Codman, D. M. D., in *Indiana Dental Journal.*

---

A NEW WAY OF PRESERVING MEAT.—A new method of preserving freshly killed meat has been discovered by a Danish zoologist, August Fjelstrup, who is the discoverer of condensing milk without the use of sugar. The system has been used in a Dutch slaughter house for three months. The animal is first shot or stunned by a shot from a revolver in such a way as not to injure the brain proper. When the animal drops down senseless, an assistant cuts down over the heart and opens a ventricle, which allows the blood to flow out, the theory of this being that the decomposition of the blood is almost entirely responsible for the quick putrefaction of fresh meats. Immediately after the blood is let out a briny solution, which varies in strength according to the time the meat is to be kept, is injected by means of a powerful syringe through the other ventricle into the veins of the body. The whole process takes only a few minutes and the beef is ready for use and can be cut up at once.—"Scientific American."

THOUGHTLESS MOTHERS.—A few words as to the health of their infant children. I am sorry to say that mothers are very thoughtless regardlng their teeth. Also regarding the masticating of food for the good of their infant children.

We hear many, many mothers exclaim that their child has such a bad stomach, and the doctor does not seem to know what is the trouble and does not give the child any relief. They don't know what to do.

If the physician would examine the teeth of the mother he might get a pointer as to the cause of the trouble. Some mothers masticate some of the food and give it to the babe to swallow, and to be digested by its little tender stomach. When the mother has several bad teeth broken down by decay, etc., it is very common for her to have pyorrhea, at least her mouth is in a bad state, even for the general health of the mother. leave alone the health of the delicate little child.

When a physician is called to attend the child he generally asks what the child has been eating. The mother generally states, "I gave it milk, potatoes, bread, meat, etc." But also states "I can't see how the meat should affect the child, as I chewed the meat all up fine before giving it to the baby to eat."

When a physician meets such cases, if he would refer the mother to their family dentist it would improve the health of the mother and child,—Claude C. Chick, M. D., D. D. S., in *Indiana Dental Journal*.

---

DENTAL DETECTION OF CRIMINALS.—Poachers were in a Yorkshire Assize Court charged with the murder of a keeper. One of the prisoners had the mark of a bite on his wrist, and an examination of the jaw of the murdered keeper showed that he had a peculiar conformation of the teeth. Plaster casts, both of the wounded wrist and of the murdered man's jaws, were made, and, the two tallying exactly, the man was convicted. In France, a man accused of the murder of a widow, had marks of bites on his right hand. The poor woman had one tooth in her upper and two in her lower jaw, and a cast made showed that these fitted exactly, and without the shadow of a doubt, into the wounds in the hand of the accused. But even more marked still was the evidence provided by dentistry at Manchester Assizes, not long since. A gentleman, who had a small dog with him, was attacked on a dark night by a ruffian who, after knocking him about, robbed him, and then promptly effected his escape. A tramp was arrested for the crime, but the prosecutor could not swear to him as the culprit, the night having been so dark, but the injured man pointed out that he had bitten his assailant on the hand, and that his little dog had also assisted with his teeth. The man arrested had marks on his hand, and he also bore marks of a dog-bite on his legs. He accounted for the latter by saying that a farmer's big dog had worried him. A dentist was, however, called in, and he showed the court and jury conclusively that the prosecutor's little dog must have produced the leg wound. The murderer of a Herr and Frau Schneider was convicted at Vienna by means of a dentist's skill and an apple which had been bitten and left near the scene of the crime. The teeth of a man suspected fitted exactly with the bitten apple. But perhaps the most singular of all these points of detection by dentists was provided by the bringing to justice of the murderer of a banker in St. Petersburg. Just near the dead body was a much worn cigar-holder containing a half-smoked cigar. From the particular excellence of the cigar itself it was supposed at first that the banker must have been smoking it, and yet this very excellence tallied but strangely with the poor quality of the cigar holder. Certain employes of the banker, to whom suspicion pointed rather vaguely, were, by the direction of a shrewd police magistrate, examined as to their teeth and jaws by a dentist, and the guilty man stood detected at once. He showed how the teeth of the accused fitted precisely into the marks on the well-worn cigar-tube.—"Dental Record."

FILLING VS. CROWN.—We should not use crowns until every possibility of filling is removed. Many bicuspids and molars that are crowned could be better preserved and more nearly approximate nature, by preparing the surface for filling, Where there is an occluding weak wall, grind it down from one-sixteenth to an eighth of an inch. Where there are no walls, insert pins in canals, allowing ends to extend up into the crown space. Take a gutta-percha cap crown, cut away the flat surface and the remaining band can be easily adjusted to the tooth, contoured with warm instruments to meet the demands. Then fill with amalgam, allowing the band to remain in situ for a day or two, when it can be removed and the amalgam polished.

There are many good molars doing excellent service, after this treatment, presenting an entire masticating surface of amalgam. Care must always be taken with amalgam to condense well and my custom is to use warm instruments in packing.—W. H. Weaver, in *Weekly*.

---

SURGICAL TREATMENT OF ABSCESS.—In opening an abscess the surface should be carefully cleansed, and other antiseptic precaution observed. All instruments should be antiseptically treated.

The first step in our operation would be cleansing of the surface with hydrogen dioxid, followed by a small injection of a five per cent. solution of eucain, an incision is then made at a point on the gum immediately overlying the apex of the affected root, with a pointed bistoury thrust down to the bone —a goodsized incision should be made. The bleeding is then encouraged by the use of hot water for a few minutes, when a pellet of cotton, which has been dipped in a solution containing one or two per cent. of cocain and antipyrin four per cent. is then laid against the periosteum at the bottom of the cut. In a few minutes bleeding will cease, when a spear-drill, driven by the engine, is passed through the bone into the tissue of the apical space.

Any bleeding which may occur is encourage. For washing incisions and the abscess there is no agent more acceptable than a twenty per cent. solution of phenol-sodique, it being both sedative and antiseptic. A fair-sized round bur is then used to cut away necrosed bone if any is found.

If it is found necessary to excise the end of a root, a small fissure-bur is used and the root rounded, leaving no rough edges.

The wound is now cauterized with a fifty per cent solution of zinc chlorid, and the cavity loosely filled with boracic acid gauze, your patient being seen every day, and less gauze inserted at each dressing as granulation progresses.

The time required for healing is from four to ten days, according to existing conditions. An antiseptic mouth-wash should be recommended in addition to the above treatment.—L. Meisberger, in *Dental Cosmos.*

---

ROOTS AND BRIDGES.—With very frail roots. you will find that when they are well cleansed mechanically of the accretions which have collected on them they will be held together by bridge-work. All roots not parallel should be ground and every bit of enamel carved off at the cervical margin, so that the crown, instead of hugging the body of the tooth, will be coneshaped, and the harder you drive it the tighter it clings. Ordinary teeth have a more offensive odor than a well-adapted bridge, for there is more secretion and more foreign particles work into the interstices.—C. L. Hungerford, in *Dental Digest.*

---

TREATMENT OF DECALCIFIED DENTINE IN BOTTOM OF CAVITY.—Apply sufficient oil of cassia to permeate all the softened dentine. Dry the surface with chloroform; fill with aristol and chloro-percha, which becomes hard and glassy when the chloroform has evaporated. Finish with cement.—R. H. Cool, in "Pacific Gazette."

IN TAKING an impression in plaster for a partial plate, or in taking a full impression of the teeth, use a clean impression cup evenly filled with plaster, leaving but little surplus. Wait till your plaster has set hard, then slip the cup off, it having been oiled slightly, leaving the plaster all in the mouth. Two grooves are then cut in the hardened plaster on a line parallel with the cuspid teeth, not cutting quite through. Then with a quick pry with a pointed spatula or knife the anterior piece is wrenched loose. The lateral pieces are broken off with the thumb and finger. The large piece covering the roof of the mouth may readily be worked loose. Then you have four pieces which are easily united.—G. D. Sitherwood, in *Illinois Society*.

---

ANNEALING GOLD.—You will never appreciate the true working qualities of cohesive gold until you quit passing it in the flame of the lamp. Use a sheet of mica or an annealing tray. Don't be penurious. The good effects will pay for the difference.—W. H. Weaver, in "Dental Weekly."

---

TO REMOVE PLASTER FROM VULCANITE PLATES.—Immerse plate for a few minutes in hydrochloric acid.—Dr. Wright, in "Pacific Gazette."

---

SOAK IT.—To cut an old model easily—soak in water a few minutes, when it will be easily trimmed.—"Dental Weekly."

